# The GALAD score and the BALAD-2 score correlate with transarterial and systemic treatment response and survival in patients with hepatocellular carcinoma

**DOI:** 10.1007/s00432-023-05526-z

**Published:** 2024-02-06

**Authors:** Anne Olbrich, Johannes Niemeyer, Hendrik Seiffert, Sebastian Ebel, Olga Gros, Florian Lordick, Dirk Forstmeyer, Daniel Seehofer, Sebastian Rademacher, Timm Denecke, Madlen Matz-Soja, Thomas Berg, Florian van Bömmel

**Affiliations:** 1 Laboratory for Clinical and Experimental Hepatology (LCEHep), Division of Hepatology, Department of Medicine II, Leipzig University Medical Center, Leipzig, Germany; 2 Department of Diagnostic and Interventional Radiology, Leipzig University Medical Center, Leipzig, Germany; 3 Department of Anesthesia and Intensive Care, Helios Clinic Köthen, Köthen, Germany; 4 University Cancer Center Leipzig (UCCL) and Division of Oncology, Department of Medicine II, Leipzig University Medical Center, Leipzig, Germany; 5 Department of Visceral, Vascular, Thoracic and Transplant Surgery, Leipzig University Medical Center, Leipzig, Germany; 6 University Liver Tumor Center (ULTC), Leipzig University Medical Center, Leipzig, Germany; 7 Rudolf-Schönheimer-Institute for Biochemistry, University of Leipzig, Leipzig, Germany; 8 Division of Hepatology, Department of Medicine II, Leipzig University Medical Center, Leipzig, Germany

**Keywords:** HCC, AFP, AFP-L3, DCP, BCLC-A/B

## Abstract

**Purpose:**

The GALAD score and the BALAD-2 score are biomarker-based scoring systems used to detect hepatocellular carcinoma (HCC). Both incorporate levels of alpha-fetoprotein (AFP), lens culinaris agglutinin-reactive AFP (AFP-L3), and des-gamma-carboxy prothrombin (DCP). Our objective was to examine the relationship between the GALAD score as well as the BALAD-2 score and treatment response to transarterial or systemic treatments in patients with HCC.

**Methods:**

A total of 220 patients with HCC treated with either transarterial (*n* = 121) or systemic treatments (*n* = 99; mainly Sorafenib) were retrospectively analyzed. The GALAD score and the BALAD-2 score were calculated based on AFP-L3, AFP, and DCP levels measured in serum samples collected before treatment. The results were correlated with 3-month treatment efficacy based on radiologic mRECIST criteria.

**Results:**

The GALAD score showed a strong correlation with BCLC stage (*p* < 0.001) and total tumor diameter before treatment (*p* < 0.001).The GALAD score at baseline was significantly lower in patients with a 3-month response to transarterial (*p* > 0.001) than in refractory patients. Among patients receiving systemic treatment, the median BALAD-2 score at baseline showed a strong association with response at month 3 (*p* < 0.001).

In the transarterial treatment group, the GALAD score (AUC = 0.715; *p* < 0.001) as well as the BALAD score (AUC = 0.696; *p* < 0.001) were associated with overall survival, hereby outperforming AFP, AFP-L3 and DCP.

**Conclusion:**

The GALAD score as well as the BALAD-2 score hold significant promise as a prognostic tool for patients with early or intermediate-stage HCC who are undergoing transarterial or systemic treatments.

**Supplementary Information:**

The online version contains supplementary material available at 10.1007/s00432-023-05526-z.

## Highlights

- The GALAD score is associated with response of HCC to transarterial treatments.
- The BALAD-2 score is associated with response of HCC to systemic treatments.
- The GALAD score is also associated with response in HCC patients with AFP levels ≤ 20 ng/mL.
- The GALAD score as well as the BALAD-2 score at baseline correlate with overall survival in patients who received transarterial therapy.

**Supplementary Information:**

The online version contains supplementary material available at 10.1007/s00432-023-05526-z.

## Introduction

Hepatocellular carcinoma (HCC) is the sixth most common malignancy and a leading cause of cancer-related death worldwide (McGlynn et al. 2021). HCC generally has a poor prognosis with a 5 year survival rate of 20–40% (Llovet et al. 2021; Jemal et al. 2017). Therapeutic options for HCC continue to evolve, and given the diversity of treatment options, a multidisciplinary approach is needed that requires the involvement of surgical, medical, radiation oncology, hepatology, and interventional radiology. The Barcelona Clinic Liver Cancer (BCLC) staging system is widely used to estimate prognosis and to allocate treatment strategies (Reig et al. 2022).

According to current treatment guidelines, patients who are no longer eligible for HCC resection or liver transplantation should receive transarterial treatments, including transarterial chemoembolization (TACE) or transarterial radioembolization (TARE) (Reig et al. 2022; EASL Clinical Practice Guidelines 2018; Marrero et al. 2018; Benson et al. 2021). In addition, there is growing evidence that transarterial treatments may be used in patients in early stages (BCLC-A or -B) to provide bridging to orthotopic liver transplantation (OLT) (EASL Clinical Practice Guidelines 2018; Patidar et al. 2022). Patients in advanced stages (BCLC-C) should preferably receive systemic therapy. However, in certain constellations, other therapeutic options are also possible in both stages, resulting in a partial overlap of treatment options across different HCC stages (EASL Clinical Practice Guidelines 2018; Marrero et al. 2018). To facilitate individual optimized treatment strategies, there is a high medical need to develop response biomarkers that can help choose between different but equivalent treatment options (EASL Clinical Practice Guidelines 2018; Parikh et al. 2023).

Currently, alpha-fetoprotein (AFP) is the only widely used biomarker for screening and surveillance of HCC; however, AFP has not been established as a treatment response marker. The combination of lens culinaris agglutinin-reactive AFP (AFP-L3) and des-gamma-carboxy prothrombin (DCP) in combination with AFP has emerged as a screening biomarker that increases the sensitivity of detecting HCC alone and in combination with AFP (Wang et al. 2020). Recently, a novel scoring system derived from **G**ender, **A**ge, AFP-**L**3, **A**FP, and **D**CP, named GALAD, was established for the detection of HCC in patients with chronic liver disease (Johnson et al. 2014). The GALAD score has been extensively validated as a sensitive method for detecting HCCs in Asian and European patient cohorts with different underlying liver diseases (Liu et al. 2020; Schotten et al. 2021; Best et al. 2020). The BALAD score, referring to **B**ilirubin, **A**lbumin, AFP-**L**3, **A**FP and **D**CP, is a model that incorporates the use ot the markers total bilirubin and albumin which are associated with unfavorable outcomes (Toyoda et al. 2006; Chan et al. 2015). The BALAD-2 score relies on a more complex statistical analysis and provides a slightly better performance as compared to the BALAD score (Fox et al. 2014; Berhane et al. 2016). At present, it is unclear whether the GALAD or BALAD-2 scores, beyond their use as a diagnostic tool, can be predictors of treatment effectiveness. To address this question, we assessed the potential role of the GALAD and BALAD-2 scores and their components AFP, AFP-L3, and DCP as biomarkers for transarterial or systemic treatment outcomes in a large European HCC patient cohort.

## Methods

### Ethics statement

The study design and all experimental procedures were approved by the Ethics Committee of the University of Leipzig (ethics committee project numbers 006-09 and 112/18-ek) and conducted in accordance with the Declaration of Helsinki.

### Human Subjects

All patients receiving transarterial or systemic treatment for HCC at the Leipzig University Medical Center between 2010 and 2019 were retrospectively screened for enrollment (*n* = 1186 patients).

The inclusion criteria were (i) treatment with either transarterial (TACE or TARE) or systemic treatment, (ii) follow-up and disease staging based on CT or MRI at 10–12 weeks after treatment initiation, (iv) age > 18 years, (v) availability of a serum sample collected at treatment initiation and stored at − 20 °C, and (vi) written informed consent. Patients were excluded if they had malignancies other than HCC, mixed hepatocellular or cholangiocarcinoma, or fibrolamellar HCC. Accordingly, 512 patients were excluded due to ineligibility for transarterial or systemic treatment, and another 454 patients were excluded due to a lack of serum samples before the start of therapy, the existence of other tumor entities, OLT, or the absence of informed consent. A total of 220 patients were analyzed in this study.

### HCC diagnosis and treatment evaluation

The diagnosis of HCC and treatment response were confirmed based on either contrast-enhanced multiphase computed tomography (CT) or magnetic resonance imaging (MRI) according to current treatment guidelines (EASL Clinical Practice Guidelines 2018). Tumor stage was defined according to the BCLC staging system (Reig et al. 2022). Treatment allocation was based on the recommendations of a multidisciplinary tumor board. Treatment efficacy was evaluated using the mRECIST criteria based on MRI 10–12 weeks after treatment initiation. Response to TACE was defined according to the concept proposed by the Japanese Society of Hepatology (JSH) in 2021 (Kudo et al. 2021). Accordingly, responders to transarterial treatments were defined as patients with complete or partial response or stable disease, while refractoriness to transarterial or nonresponse to systemic treatment was defined as progressive disease, viable lesion > 50%, tumor revascularization, appearance of new hypervascularized intrahepatic lesions, or increased vascular invasion (Kudo et al. 2021, 2014; Llovet and Lencioni 2020).

### HCC biomarker quantification

AFP, AFP-L3, and DCP were measured in the serum by Fujifilm Wako Chemicals Europe (Neuss, Germany). The lower detection limits were 0.03 ng/mL, 0.6% and 0.17 ng/mL, respectively.

### Calculation of the GALAD score

The GALAD score was calculated according to the equation (*Z* =  − 10.08 + 0.09 × age + 1.67 × gender + 2.34 × log10 AFP (ng/mL) + 0.04 × AFP-L3 (%) + 1.33 × log10 DCP (ng/mL)). Gender was defined as 1 for males and 0 for females (Johnson et al. 2014).

### Calculation of the BALAD-2 score

The BALAD-2 function was calculated using the following equation: Linear predictor (xb) = 0.02 * [AFP (ng/mL) − 2.57] + 0.012 * [AFP-L3 (%) − 14.19] + 0.19 * [ln(DCP (ng/mL)] − 1.93) + 0.17 * {[bilirubin (μmol/L)^1/2^] − 4.50} − 0.09 * [ALB (g/L) − 35.11] (Fox et al. 2014).

### Statistics

IBM SPSS Statistics software version 25 was used for data analyses. A *p*-value less than 0.05 was considered significant. For the description of continuous variables, mean and standard deviation or median and interquartile range were used as appropriate, whereas for the description of qualitative variables, absolute frequencies and percentages were used. Differences between two independent groups were tested using the Mann–Whitney *U* test. Pearson’s correlation coefficients were used to calculate correlations between variables. Receiver operating characteristic (ROC) curves were constructed to assess sensitivity, specificity, and respective areas under the curves (AUCs) with 95% confidence intervals (CI). The time point before the start of transarterial or systemic treatment was defined as baseline. To determine the best cut-off point for therapy response, the highest Youden’s index was calculated.

## Results

### Patient selection and baseline characteristics

A total of 1186 patients were assessed for inclusion into this retrospective study and 220 patients (mean age 65.0 ± 9.2 years [31–84 years], 190 males), including 121 patients with transarterial and 99 patients with systemic treatment, were analyzed (Fig. S1).

At the start of treatment, 194 patients (88%) had liver cirrhosis in Child–Pugh stages A (*n* = 150; 77%), B (n = 39; 20%), and C (*n* = 5; 3%) (Table 1). The etiology of the underlying liver disease was mainly alcoholic (*n* = 111; 57%), followed by viral hepatitis (*n* = 34; 18%), and NASH (*n* = 21; 11%). A total of 47 (21.3%) patients were classified as BCLC-A, 113 (51%) as BCLC-B, and 61 (28%) as BCLC-C. The majority of HCC patients (79%) were diagnosed with an advanced stage (B and C) according to the BCLC staging system. Patients with BCLC-A received transarterial treatment as bridging therapy for OLT. Most patients in the systemic treatment group received sorafenib (n = 70; 71%; Table S1). Twenty-six patients received systemic treatment, without prior loco-regional treatment (Table S2).

**Table 1:** Characteristics of the patient population at baseline

Characteristics, n (%)	Transarterial treatment (*n* = 121)	Systemic treatment (*n* = 99)	*p*-value
Sex (male)	105 (87)	85 (86)	0.825
Age at baseline (years)	63.7 ± 9.0	66.6 ± 9.3	0.011
Liver cirrhosis	109 (90)	85 (86)	0.335
Viral hepatitis	19 (17)	15 (18)	
Alcohol-related cirrhosis	64 (59)	47 (55)	
NASH	11 (10)	10 (12)	
others	15 (14)	13 (15)	
Child–Pugh-Turcotte classification			0.629
A	86 (79)	64 (75)	
B	19 (17)	20 (24)	
C	4 (4)	1 (1)	
BCLC stages			< 0.001
A	46 (47)	0 (0)	
B	61 (50)	52 (53)	
C	14 (12)	47 (47)	
Number of tumor lesions			0.019
1	55 (45)	2 (2)	
2	28 (23)	2 (2)	
3	14 (12)	3 (3)	
> 3	26 (21)	5 (5)	
n.a	0 (0)	87 (88)	
Total tumor diameter at baseline			< 0.001
< 3 cm	28 (23)	1 (1)	
3–5 cm	40 (33)	9 (9)	
6–10 cm	36 (30)	17 (17)	
> 10 cm	17 (14)	15 (15)	
n.a	0 (0)	57 (58)	

*BCLC*  Barcelona clinic liver cancer, *n.a.* not available

### Treatment response and survival

In patients receiving transarterial treatment, the response rate after 10–12 weeks was 71% (86/121) and the median overall survival (OS) was 13 [0–89] months. In patients receiving systemic treatment, the response rate at week 12 was 31% (31/99) and the median OS was 9 [0–57] months. In the total study population, the overall response after three months was 53% (117/220), and the median overall survival was 11 [0–89] months (Fig. S1).

### Association between the GALAD score and and the BALAD-2 score with tumor and patient characteristics

**Fig. 1:**
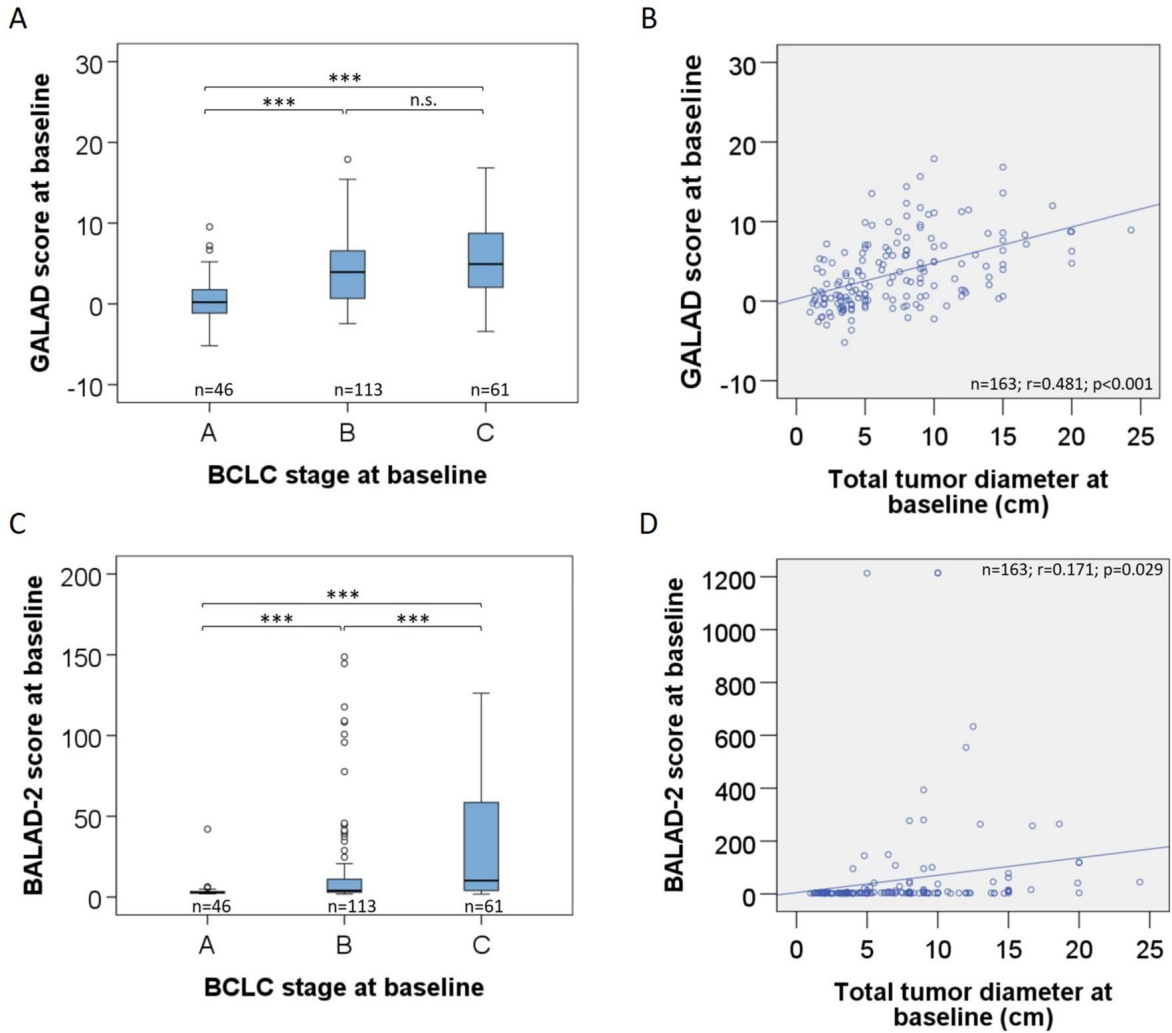
Association of the GALAD score and the BALAD-2 with tumor size and distribution at baseline. **A** The GALAD score was significantly lower in patients in BCLC stage A than in those in BCLC stage B or C. The upper and lower ends of the bar indicate the 75th and 25th percentiles, respectively. The marking in the middle of the bar shows the median. **B** Intermediate correlation of the total HCC diameter by tomography imaging and the GALAD score at baseline (scatter plot). The line represents linear regression. **C** The BALAD-2 score was significantly lower in patients in BCLC stage A than in those in BCLC stage B or C. The upper and lower ends of the bar indicate the 75th and 25th percentiles, respectively. The marking in the middle of the bar shows the median. **D** Intermediate correlation of the total HCC diameter by tomography imaging and the BALAD-2 score at baseline (scatter plot). The line represents linear regression. *** = *p* < 0.001 (Mann–Whitney U test); *n.s.* = not significant; *n* = number of patients; *r* = correlation coefficient of *r*

The GALAD score at baseline ranged from − 5.19 to 17.89. The mean baseline GALAD scores were significantly higher in patients with BCLC stages B or C than in those with BCLC stage A (*p* < 0.001 and *p* < 0.001, respectively) (Fig. 1a). The GALAD score as well as the levels of AFP, AFP-L3, and DCP were significantly higher in patients receiving systemic treatments than in those receiving transarterial treatment according to the different distribution of BCLC stages in these patient populations (Table 2). Moreover, across the entire study population, the GALAD score moderately correlated with the total tumor diameter before treatment (*r* = 0.481; *p* < 0.001) (Fig. 1b).

**Table 2:** Median serum levels at baseline of AFP, AFP-L3 and DCP and median GALAD score of the total patient population and of the patient groups receiving transarterial or systemic treatments

Biomarker	Total patient population (*n* = 220)	Transarterial treatment (*n* = 121)	Systemic treatment (*n *= 99)	*p*-value
AFP	36.9 [1–60500] ng/mL	17.3 [1–60500] ng/mL	86.7 [2–60500] ng/mL	0.002
AFP-L3	10.4 [0–93.7] %	8.9 [0–87.1] %	13.5 [0–93.7] %	0.03
DCP levels	12.7 [0–9721] ng/mL	6.1 [0–9721] ng/mL	27.8 [0–3201] ng/mL	0.008
GALAD score	3.89 [−5.19–17.89]	1.9 [−5.19–17.89]	4.63 [−3.42–16.83]	< 0.001
BALAD-2 score	3.91 [1.79–1215]	3.49 [1.83–1215]	5.76 [1.79–1214]	< 0.001

No significant differences were found between females (*n* = 30) and males (*n* = 190) regarding the levels of AFP (*p* = 0.253), AFP-L3 (*p* = 0.381), and DCP (*p* = 0.989), as well as age at treatment initiation (*p* = 0.512) and the GALAD score (*p* = 0.537). The levels of AFP (*p* = 0.661), AFP-L3 (*p* = 0.756), and DCP (*p* = 0.605) as well as the GALAD score (*p* = 0.499) were similar between patients with and without liver cirrhosis (Table 3). There was no overall influence of underlying liver disease on the GALAD score (*p* = 0.342). However, patients with NASH-related liver cirrhosis showed higher GALAD scores than patients with alcoholic cirrhosis (*p* = 0.05).

**Table 3:** Median serum levels at baseline of AFP, AFP-L3 and DCP and median GALAD score in females and males and in patients with and without cirrhosis

Biomarker	Females (*n* = 30) versus males (*n* = 190)	No cirrhosis (*n* = 26) versus liver cirrhosis (*n* = 194)
AFP	85.7 [2–31495] ng/mL versus 29.0 [1–60500] ng/mL *p* = 0.253	64.4 [1–60500] ng/mL versus 35.9 [2–60500] ng/mL *p* = 0.661
AFP-L3	15.0 [0–92.8] % versus 10.2 [0–93.7] % *p* = 0.381	13.8 [0–92.8] % versus 10.2 [0–93.7] % *p* = 0.756
DCP	13.1 [0–3201] ng/mL versus 12.7 [0–9721] ng/mL *p* = 0.989	35.3 [0–6367] ng/mL versus 12.9 [0–9721] ng/mL *p* = 0.605
GALAD score	2.17 [−3.65–13.04] versus 3.18 [−5.19–17.89] *p* = 0.537	4.45 [−3.65–17.89] versus 2.97 [−5.19–16.83] *p* = 0.499
BALAD-2 score	5.11 [1.79–633] versus 3.82 [1.83–1215] *p* = 0.358	4.46 [1.79–1215] versus 3.87 [1.96–1214] *p* = 0.898

The BALAD-2 scores at baseline spanned a wide range from 1.79 to 1215. Mean baseline BALAD-2 score results showed a linear increase across to BCLC stages A–C (p < 0.001) with stronger differences between BCLC-B and -C as compared to the GALAD score (Fig. 1c). Furthermore, the BALAD-2 score was notably elevated in patients undergoing systemic treatments when compared to those undergoing transarterial treatments (Table 2). Across the entire study cohort, there was a weaker correlation between the BALAD-2 score and the total tumor diameter before treatment (*r *= 0.171; *p* = 0.029) as compared to the GALAD score (Fig. 1d). BALAD-2 score results were similar in male or female patients (*p* = 0.358) and in patients with and without liver cirrhosis (*p* = 0.898) (Table 3) and showed no correlation with age at treatment initiation (*p* = 0.229). There was no overall influence of underlying liver disease on the BALAD-2 score (*p* = 0.538).

### Association of GALAD and the BALAD-2 scores with response to transarterial treatment

**Fig. 2:**
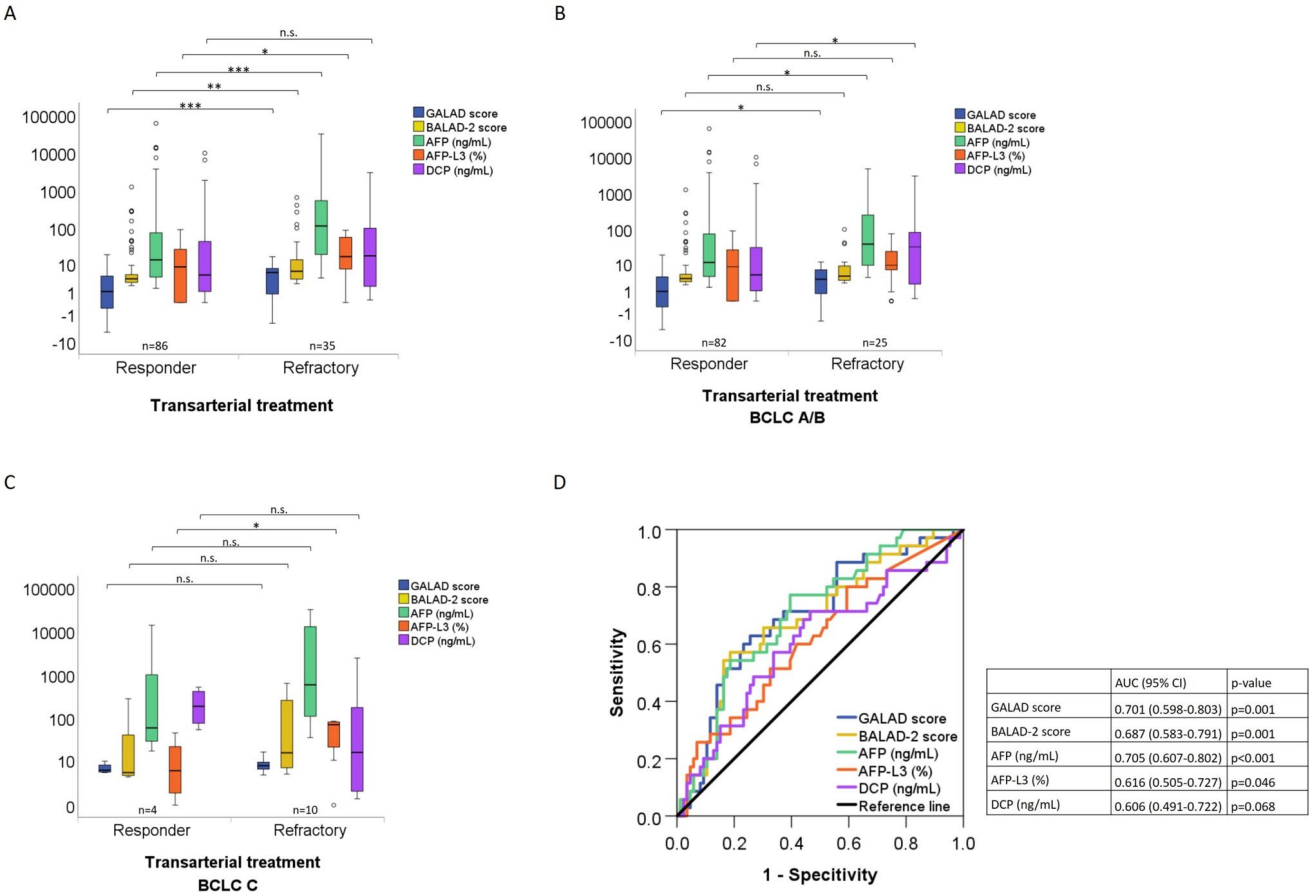
Association of the GALAD score, the BALAD-2 score, AFP, AFP-L3, and DCP the 3-month response to transarterial treatment. **A** The GALAD score, the BALAD-2 score and serum levels of AFP, AFP-L3, and DCP at baseline were grouped by response to transarterial treatment. The upper and lower ends of the bar indicate the 75th and 25th percentiles, respectively. The marking in the middle of the bar shows the median. **B** The GALAD score, the BALAD-2 score and serum levels of AFP, AFP-L3, and DCP at baseline were grouped by response to transarterial treatment in patients with BCLC-A/B. The upper and lower ends of the bar indicate the 75th and 25th percentiles, respectively. The marking in the middle of the bar shows the median. **C** The GALAD score, the BALAD-2 score and serum levels of AFP, AFP-L3, and DCP at baseline were grouped by response to transarterial treatment in patients with BCLC-C. The upper and lower ends of the bar indicate the 75th and 25th percentiles, respectively. The marking in the middle of the bar shows the median. **D** Performance of the GALAD model and the BALAD-2 score for response to transarterial treatment. * = *p* < 0.05; ** = *p* < 0.01; *** = *p* < 0.001 (Mann–Whitney *U* test); *n.s.* = not significant; *n* = number of patients

The median GALAD score at baseline was significantly lower in patients with a 3-month response to transarterial treatment than in refractory patients (0.97 versus 5.32; *p* < 0.001) (Fig. 2a). Within the group of BCLC-A/B patients, the median GALAD score before transarterial treatment was significantly lower in patients with a 3-month response than in those with refractory disease (*p *= 0.03). However, in the BCLC-C group, the GALAD score was similar in month 3 responders and refractory patients (Fig. 2b–c**)**. Similar to the GALAD score, the median BALAD-2 score at baseline was significantly lower in patients with a 3 month-response to transarterial treatment than in refractory patients (3.31 versus 5.83; *p* = 0.001) (Fig. 2a). However, BALAD-2 score results did not differ by 3 month response to transarterial treatment between BCLC-A/B patients (3.20 versus 3.91; *p* = 0.068) (Fig. 2b) or BCLC-C patients 4.63 versus 15.06; *p* = 0.157) (Fig. 2c). In the overall transarterial treated patient group the optimum cut-off for the BALAD-2 score for response to transarterial treatment was 5.28 with a sensitivity of 57% and a specificity of 81%.

In the total population of patients receiving transarterial treatment, the GALAD score had a similar association with the response to transarterial treatment (AUC = 0.701; 95% CI (0.598–0.803)) as compared to the BALAD-2 score (AUC = 0.687; 95% CI (0.583–0.791) and AFP (AUC = 0.705; 95% CI (0.607–0.802)). In contrast, AFP-L3 showed a less positive association (AUC = 0.616; 95% CI (0.505–0.727) and DCP (AUC = 0.606; 95% CI (0.491–0.722) was not associated with response (Fig. 2d). The optimum cut-off for the GALAD score for response to transarterial treatment was 3.95 with a sensitivity of 63% and a specificity of 74%.

### Association of GALAD and BALAD-2 scores with of response to systemic treatments

**Fig. 3:**
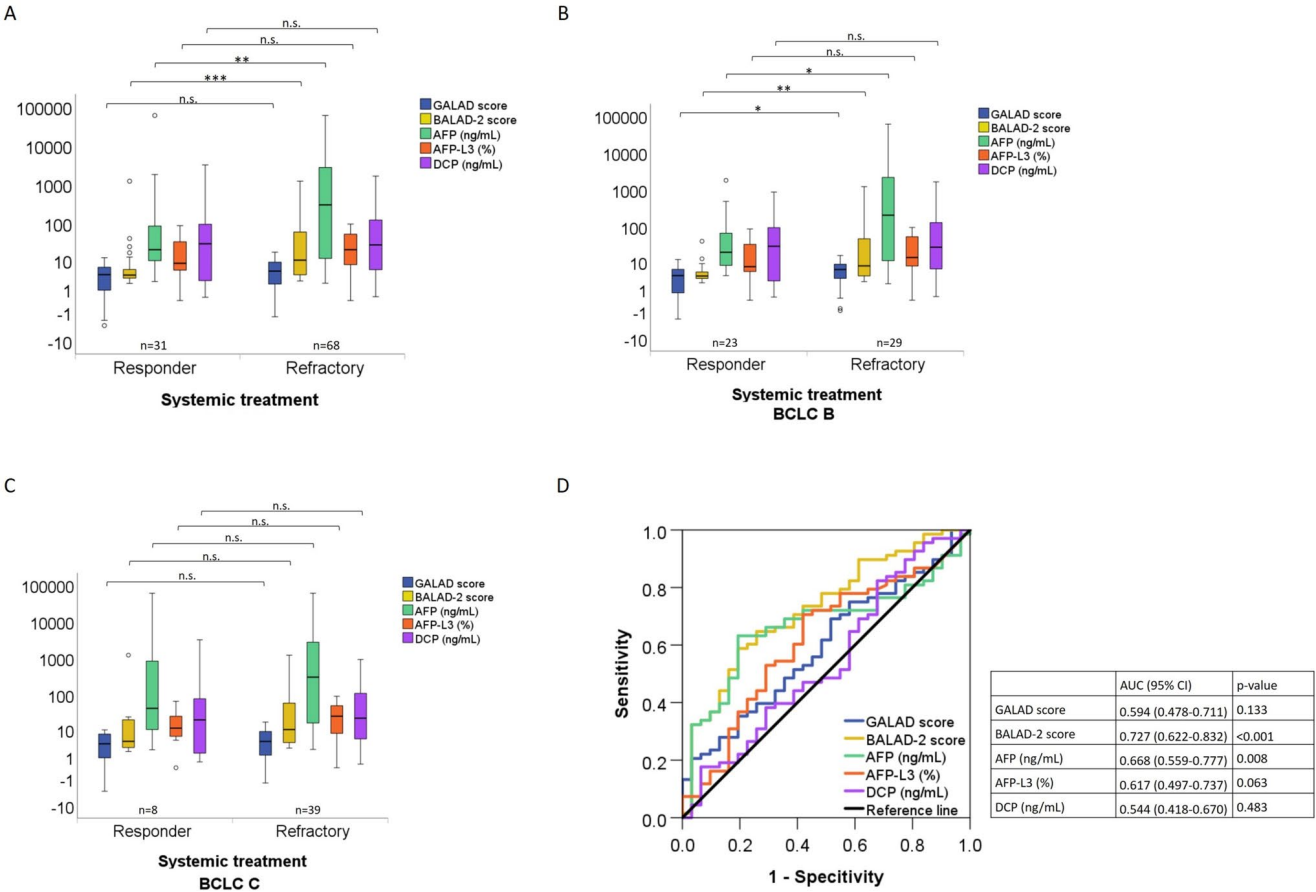
Association of the GALAD score, AFP, AFP-L3, and DCP the 3 month response to systemic treatment. **A** The GALAD score, the BLAD-2 score and serum levels of AFP, AFP-L3, and DCP at baseline were grouped by response to systemic treatment. The upper and lower ends of the bar indicate the 75th and 25th percentiles, respectively. The marking in the middle of the bar shows the median. **B** The GALAD score, the BLAD-2 score and serum levels of AFP, AFP-L3, and DCP at baseline were grouped by response to systemic treatment in patients with BCLC-B. The upper and lower ends of the bar indicate the 75th and 25th percentiles, respectively. The marking in the middle of the bar shows the median. **C** The GALAD score, the BALAD-2 score and serum levels of AFP, AFP-L3, and DCP at baseline were grouped by response to systemic treatment in patients with BCLC-C. The upper and lower ends of the bar indicate the 75th and 25th percentiles, respectively. The marking in the middle of the bar shows the median. **D** Performance of the GALAD model and the BLAD-2 score for response to systemic treatment. * = *p *< 0.05; ** = *p* < 0.01; *** = *p *< 0.001 (Mann–Whitney *U* test); *n.s.* = not significant; *n* = number of patients

The median GALAD score at baseline was similar in patients with a 3-month response to systemic treatment compared to nonresponders (3.68 versus 4.77; *p* = 0.133) (Fig. 3a). However, within the group of BCLC-B, the median GALAD score before systemic treatment was significantly lower in patients with a 3-month response than in those without (*p* = 0.05) (Fig. 3b). In contrast, the GALAD score was similar between month 3 responders and nonresponders in the BCLC-C group (Fig. 3c). In patients with BCLC-B, the GALAD score had a similar association with treatment response (AUC = 0.660; 95% CI (0.510–0.809)) as AFP (AUC = 0.667; 95% CI (0.516–0.818)) and a stronger association with response to HCC treatment compared to AFP-L3 (AUC = 0.618; 95% CI (0.462–0.774)) and DCP (AUC = 0.550; 95% CI (0.390–0.710)) (Fig. 3c). The optimum cut-off for the GALAD score for response to systemic treatment in patients with BCLC-B was 4.71, with a sensitivity of 69% and a specificity of 65%. In addition to the differences in the GALAD score, there were lower levels of AFP in month 3 responders compared to nonresponders in BCLC-B patients (*p* = 0.04) (Fig. 3a). All markers had similar levels in month 3 responders and nonresponders among BCLC-C patients (Fig. 3b).

In contrast to the GALAD score, the median BALAD-2 score at baseline was significantly lower in patients with a 3 month-response to systemic treatment than in refractory patients (3.55 versus 10.08; *p *< 0.001) (Fig. 3a). Within the group of BCLC-B, the median BALAD-2 score before systemic treatment was significantly lower in patients with a 3 month response than in those without (*p* = 0.003) (Fig. 3b). However, the BALAD-2 score was similar between month 3 responders and nonresponders in the BCLC-C group (Fig. 3c).

In the overall cohort treated with systemic drugs, the BALAD-2 score had the strongest association with response (AUC = 0.727; 95% CI (0.622–0.832)) followed by AFP (AUC = 0.668; 95% CI (0.559–0.777)). The GALAD score, AFP-L3 and DCP had no association with response in this cohort (Fig. 3d). The optimum cut-off for the BALAD-2 score for response to systemic treatment was 6.62, with a sensitivity of 59% and a specificity of 81%.

### Association of the GALAD score and the BALAD-2 score with 3-month response in patients with AFP levels ≤ 20 ng/mL

**Fig. 4:**
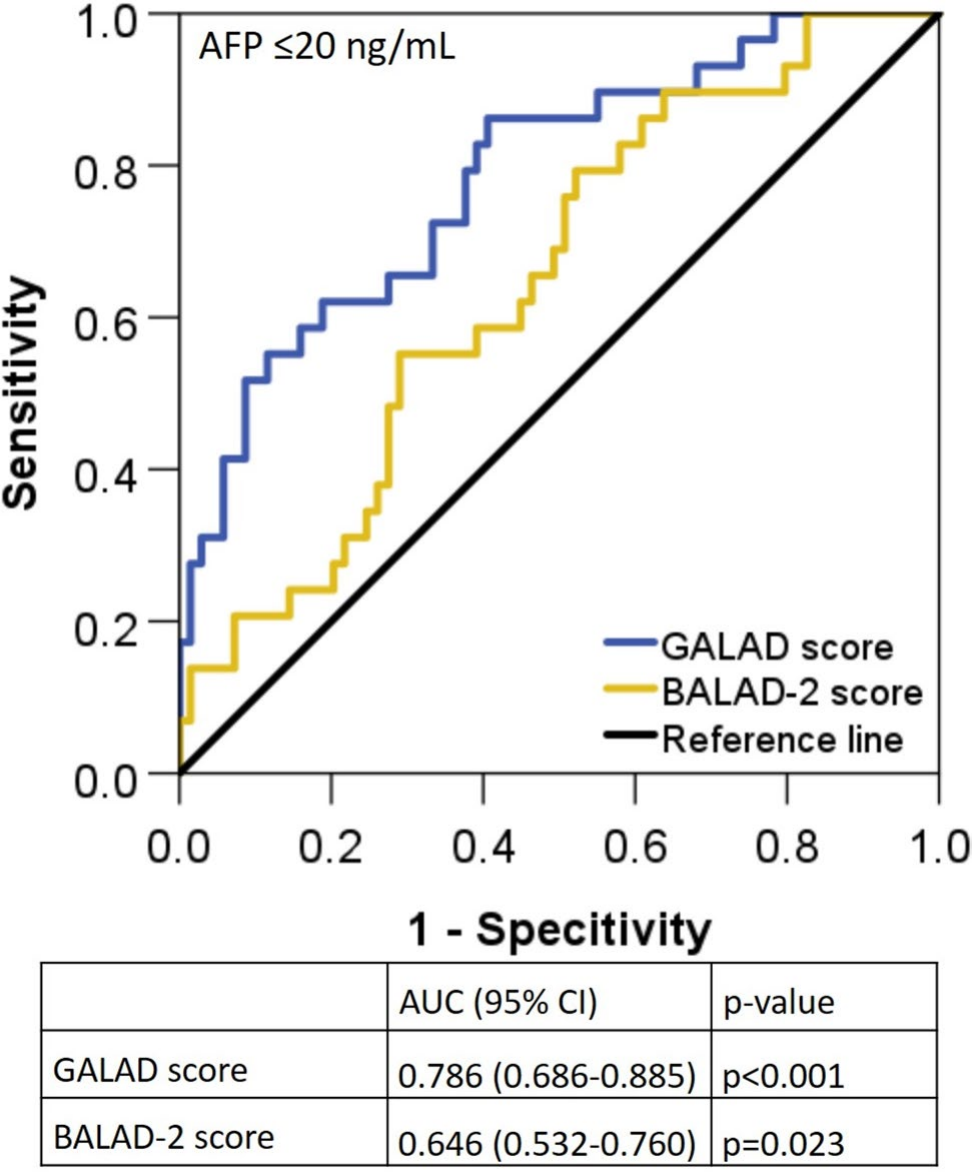
Association of the GALAD score and the BALAD-2 score with 3-month response in patients with AFP levels ≤ 20 ng/mL

In the overall cohort 98 patients showed AFP levels ≤ 20 ng/mL (45%). The association of the GALAD score and the BALAD-2 score with response was analyzed in this patient subgroup. Among these patients, the median GALAD scores in responders (*n* = 69) and refractory patients/nonresponders (*n* = 29) were 0.32 [− 5.19–6.57] versus 2.94 [− 1.06–15.42] (*p* =  < 0.001), and GALAD score results identified responders with an AUC of 0.786 (95% CI (0.686–0.885); *p* < 0.001) (Fig. 4a). The median BALAD-2 scores in responders and refractory patients/nonresponders were 2.67 [1.79–4.04] versus 3.04 [2.18–4.13] (*p* = 0.023), and BALAD-2 score results identified responders with an AUC of 0.646 (95% CI (0.532–0.760); *p* = 0.023) (Fig. 4a).

### Association of the GALAD and the BALAD-2 score with overall survival

**Fig. 5:**
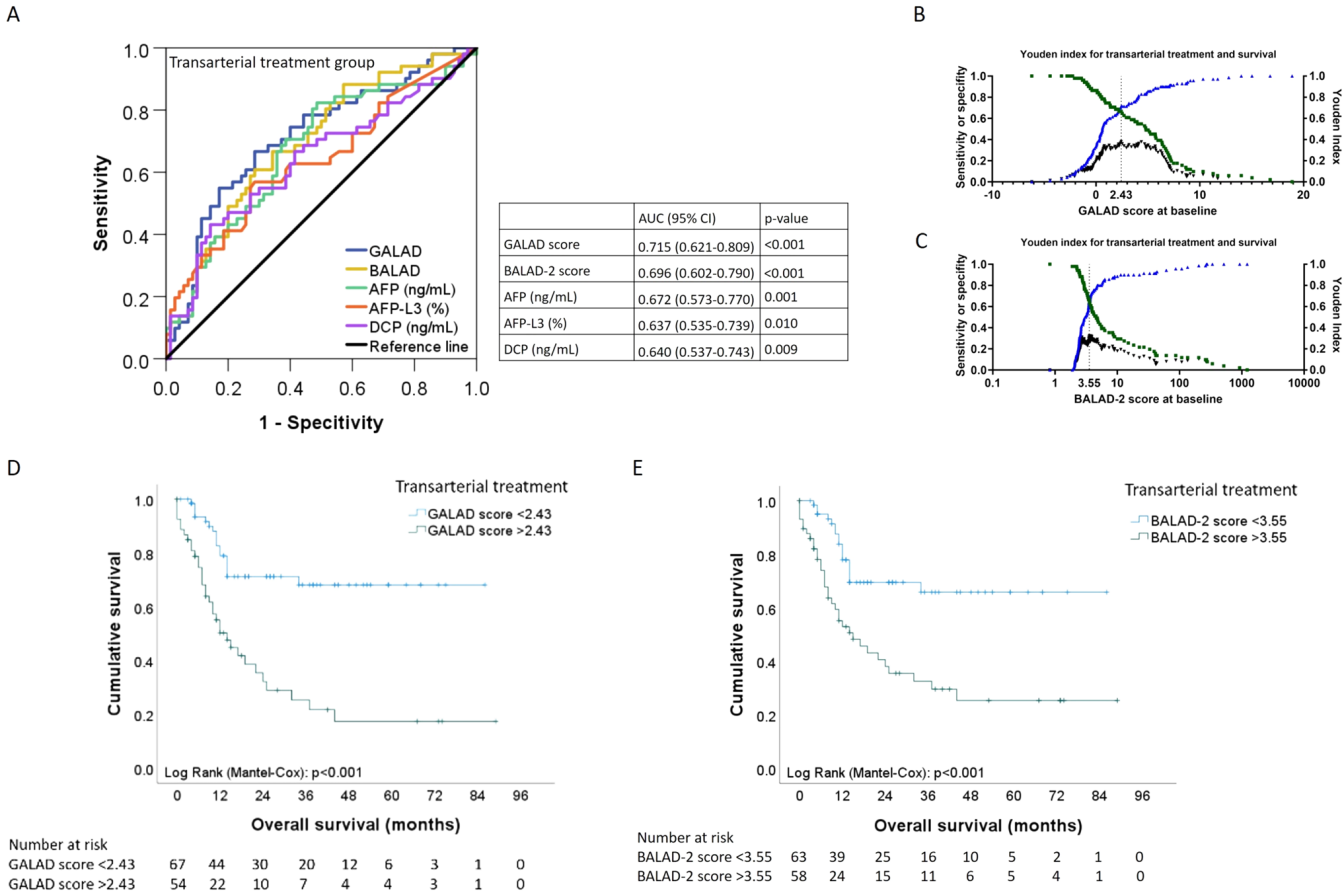
Association of the GALAD score and the BALAD-2 score with survival. **A** Performance of the GALAD model and the BALAD-2 score for survival in patients with transarterial treatment. **B** Graphical display of the optimum survival cut-off for the GALAD score at 2.43. **C** Graphical display of the optimum survival cut-off for the BALAD-2 score at 3.55. **D** Survival analysis of the patient group with transarterial treatment (*n* = 121). Patients with a GALAD score < 2.43 (*n *= 67) showed better survival than patients with a higher GALAD score at baseline (n = 54). **E** Survival analysis of the patient group with transarterial treatment (*n* = 121). Patients with a BALAD.2 score < 3.55 (*n* = 63) showed better survival than patients with a higher GALAD score at baseline (*n* = 58)

In the transarterial treatment group, the GALAD score (AUC 0.715 (95% CI (0.621–0.809); *p* < 0.001) as well as the BALAD score (AUC = 0.696; 95% CI (0.602–0.790); *p* < 0.001) were associated with overall survival;hereby outperforming AFP, AFP-L3 and DCP (Fig. 5a). The optimum cut-off for the GALAD score to split the overall survival time was 2.43 with a sensitivity of 67% and a specificity of 71%. Accordingly, patients with a GALAD score < 2.43 (Fig. 5b) showed a median survival of 17 [1–86] months, while patients with a GALAD score > 2.43 had a median survival of 10 [0–89] months (*p* < 0.001; Fig. 5d). The optimum cut-off for the BALAD-2 score to split the overall survival time was 3.55 with a sensitivity of 67% and a specificity of 66% (Fig. 5c). Patients with a BALAD-2 score < 3.55 showed a median survival of 14 [3–86] months, while patients with a BALAD-2 score > 3.55 had a median survival of 9.5 [0–89] months (*p* < 0.001) (Fig. 5e).

In contrast, in the patient group with systemic treatment, whether the GALAD score (AUC = 0.418; 95% CI (0.301–0.534); *p* = 0.169) nor the BALAD-2 score (AUC = 0.522; 95% CI (0.402–0.642); *p *= 0.769) were associated with overall survival (Fig. S2).

## Discussion

The global incidence of HCC is increasing, and the majority of HCC patients are diagnosed at intermediate or advanced stages when curative treatments are no longer indicated (Ahmed et al. 2021). There is a high medical need for biomarkers that can identify the most promising treatment options. In the present study, we demonstrated that both the GALAD and the BALAD-2 score have the potential to be a prognostic response marker for European HCC patients undergoing the most common treatment approaches, which include transarterial or systemic therapies. Thus, the BALAD-2 score could identify responders to transarterial treatment similar to the GALAD score (AUC = 0.68 versus 0.7) and was superior to the GALAD score in identifying month 3 responders to systemic treatment (AUC = 0.72 versus 0.59). Using a cut-off of 2.43, the GALAD score could identify patients in stages BCLC-A, -B, or -C with long survival following transarterial treatment. The BALAD-2 score could identify long-term survivors with a cut-off of 3.55. Our study is the first to demonstrate that the GALAD score and the BALAD-2 score have high potential as a decision-making tools for the treatment of HCC.

The GALAD score has been validated for early HCC diagnosis, including very large cohorts and early stages (BCLC-0/A) of various etiologies, prognosis prediction, and risk monitoring of HCC (Schotten et al. 2021; Best et al. 2020; Johnson et al. 2014; Berhane et al. 2016; Toyoda et al. 2021; Huang et al. 2022). Notably, in a recent phase III study, the GALAD score was associated with improved sensitivity for HCC detection but an increase in false-positive results (Tayob et al. 2023). Due to the high specificity of DCP and AFP-L3 for HCC, investigation of the association between the GALAD score and response to antitumor therapy seems obvious. Indeed, the serum levels of DCP and AFP-L3 alone were shown to be associated with the response of HCC to loco-regional treatments and survival in a large Asian cohort (Hiraoka et al. 2019). The BALAD-2 score was shown to reliably indicate the prognosis of HCC patients irrespective of etiology and cancer size, hereby providing a modest improvement in prognostic performance over the original BALAD model across all stages of disease and all etiologies (Berhane et al. 2016). However, the association between the GALAD score or the BALAD-2 score, respectively, and response to HCC treatment has not yet been investigated.

In our study, the GALAD score before initiation of transarterial treatment was significantly lower in patients showing response at month 3 as compared to refractory patients (Table 2**, **Fig. 2a). Similarly, the GALAD score before systemic treatment initiation was higher in patients showing no response at 3 months of treatment (Table 2**, **Fig. 3a). This association could be related to correlation of the GALAD score to the tumor size (Fig. 1b) and possibly to the differentiation grading of HCCs. The GALAD score showed a similar performance in identifying patients with response to transarterial treatment at month 3 (AUC = 0.64) or systemic treatments (AUC = 0.66) (Fig. 3a**, **Fig. 3b). In both patient groups, the performance of the GALAD score for classifying responders was similar to that of AFP alone but superior to that of AFP-L3 and DCP (Fig. 2c**, **Fig. 3c). Interestingly, the GALAD score and BALAD-2 score were also associated with response in patients with AFP levels ≤  20 ng/mL (AUC = 0.747 (95% CI (0.623–0.871); *p* = 0.001)) (Fig. 4). This observation merits particular attention because there are currently no alternative serum response markers available for patients with normal AFP levels. There was no association between the GALAD score or its components and response in BCLC-C patients in either treatment cohort, with the exception of AFP-L3 in patients receiving transarterial treatment (Fig. 2c**, **Fig. 3c). This seems plausible, as the BCLC-C stage is characterized by tumor spread into the blood vessels, lymph nodes, or other body organs. With the BALAD-2 score, the identification of responders at month 3 to either transarterial or systemic treatments was similar or even better than with the GALAD score in our patient population (Fig. 2d**, **Fig. 3d). Of note, the BALAD-2 score could not identify responders to systemic within the subgroups of patients in BCLC-B or -D, an observation that may be associated with the weak correlation of the BALAD-2 score to the total tumor diameter (Fig. 1d) and its inclusion of parameters reflecting liver function.

In the patient population receiving transarterial treatment, the GALAD and the BALAD-2 scores were similarly associated with OS (Fig. 5a). We were able to define a GALAD score of 2.43 and a BALAD-2 score of 3.55 as the optimal separators for patients with a high or low OS (Fig. 5d**, **Fig. 5e). The slightly better performance of the BALAD-2 score might be associated with its inclusion of liver function parameters which play a key role for survival in patients with HCC. Also, the AFP value alone showed association with OS (Fig. 5a). Indeed, it has previously been shown that in nonsurgical interventional approaches to HCC treatment, pre-intervention AFP correlates with survival (Cerban et al. 2018); however, AFP has not yet been validated as a response prediction marker (Colli et al. 2021; Toader et al. 2019). Interestingly, the GALAD score was not useful for estimating overall survival of patients receiving systemic treatment (Figure S2). Regarding the correlation of GALAD results before transarterial treatment and survival in our patients, it needs to be taken into account that survival after TACE can be influenced by multiple factors, such as sequential therapies. It is likely that the lack of benefit of the GALAD and the BALAD-2 scores is due to the different nature of therapies in the two groups and tumor biology, but this needs to be substantiated by further studies.

A limitation of our study is its retrospective design and the heterogeneity regarding different treatment approaches. The mean overall survival in the patient population receiving transarterial treatment was 13 [0–89] months, and in the systemic treatment population, it was 9 [0–57] months, which is shorter than that reported in the literature (EASL Clinical Practice Guidelines 2018). This, and the fact that some treatment allocations in our cohort are not in line with treatments suggested according to BCLC scores, are caused by the real-world characteristics of our population. Nevertheless, we demonstrated a linear increase of the GALAD score, and even more pronounced of the BALAD-2 score across BCLC stages A-C (Fig. 1a), as well as an intermediate correlation of the GALAD score with the total tumor diameter within the total study population before treatment (Fig. 1b). A similar association with HCC size has previously been described for DCP and AFP-L3 but not AFP (Sagar et al. 2021; Sauzay et al. 2016). Our observations further support the relationship between the GALAD score and disease progression and/or tumor biology. A prospective study to validate the GALAD score and the BALAD-2 score in patients receiving current first-line treatment regimens as atezolizumab and bevacizumab or tremelimumab and durvalumab, as well as standardized transarterial treatments, will be necessary to clarify the potential of the scores as response markers.

In conclusion, we could show evidence that the GALAD score and the BALAD-2 score have high potential as biomarkers for treatment response and for survival in patients with early- or intermediate stage HCCs, also in patients with low AFP levels. To further define the role of those scoring systems in clinical practice, our findings need to be validated in larger patient populations and prospective clinical trials.

### Supplementary Information

Below is the link to the electronic supplementary material.

Supplementary file1 (TIF 114 KB)

Supplementary file2 (JPG 208 KB)

Supplementary file3 (DOCX 578 KB)

## Data Availability

The data that support the findings of this study are available on request from the corresponding author. The data are not publicly available due to privacy or ethical restrictions.
