# Supplementary figures and images for: The GALAD score and the BALAD-2 score correlate with transarterial and systemic treatment response and survival in patients with hepatocellular carcinoma
Source: J Cancer Res Clin Oncol. 2024 Feb 6;150(2):81. doi: 10.1007/s00432-023-05526-z (PMC10847183; doi:10.1007/s00432-023-05526-z)

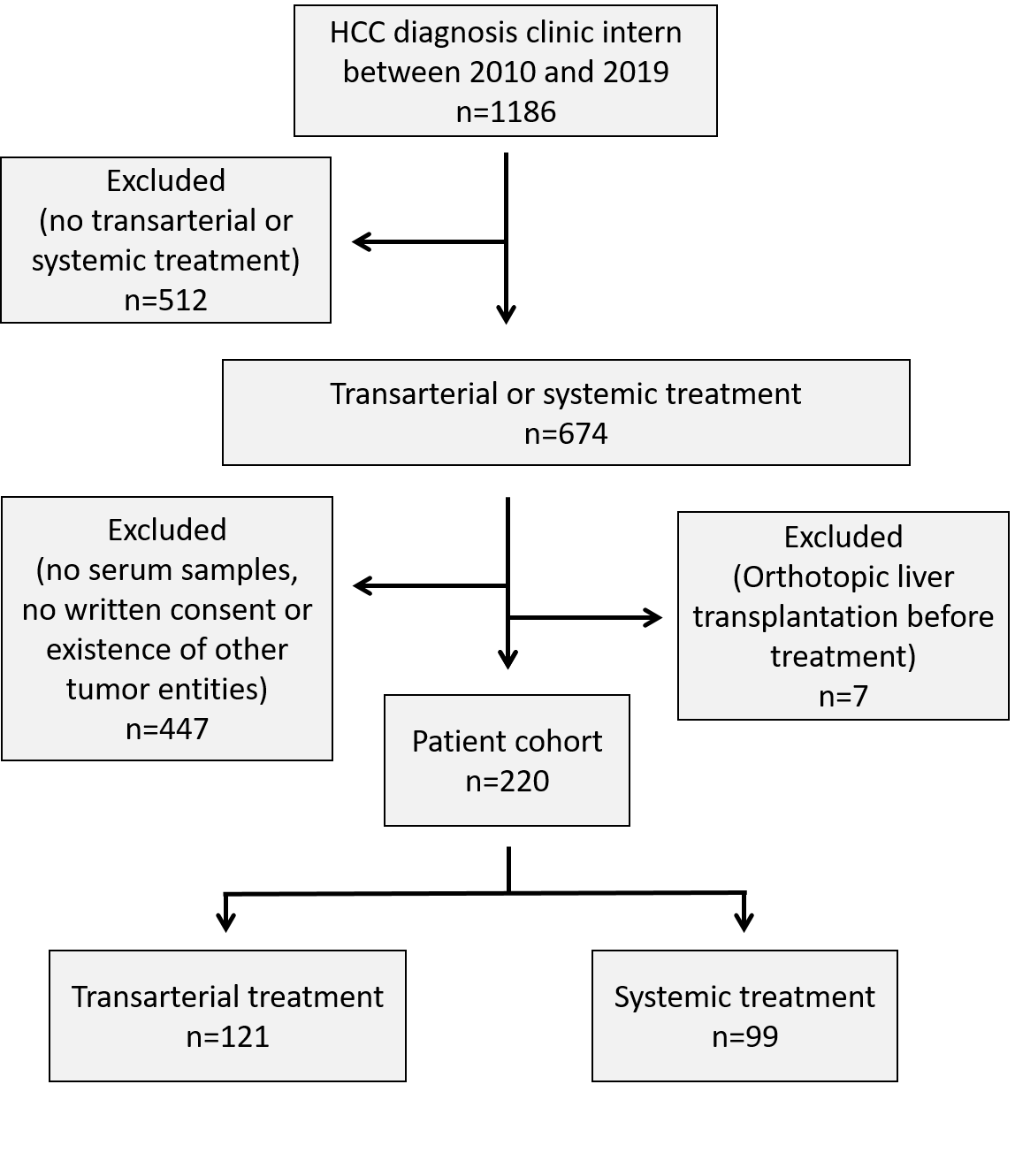

Supplement: Supplementary file 1 — Supplementary file1 (TIF 114 KB) [file 432_2023_5526_MOESM1_ESM.tif]

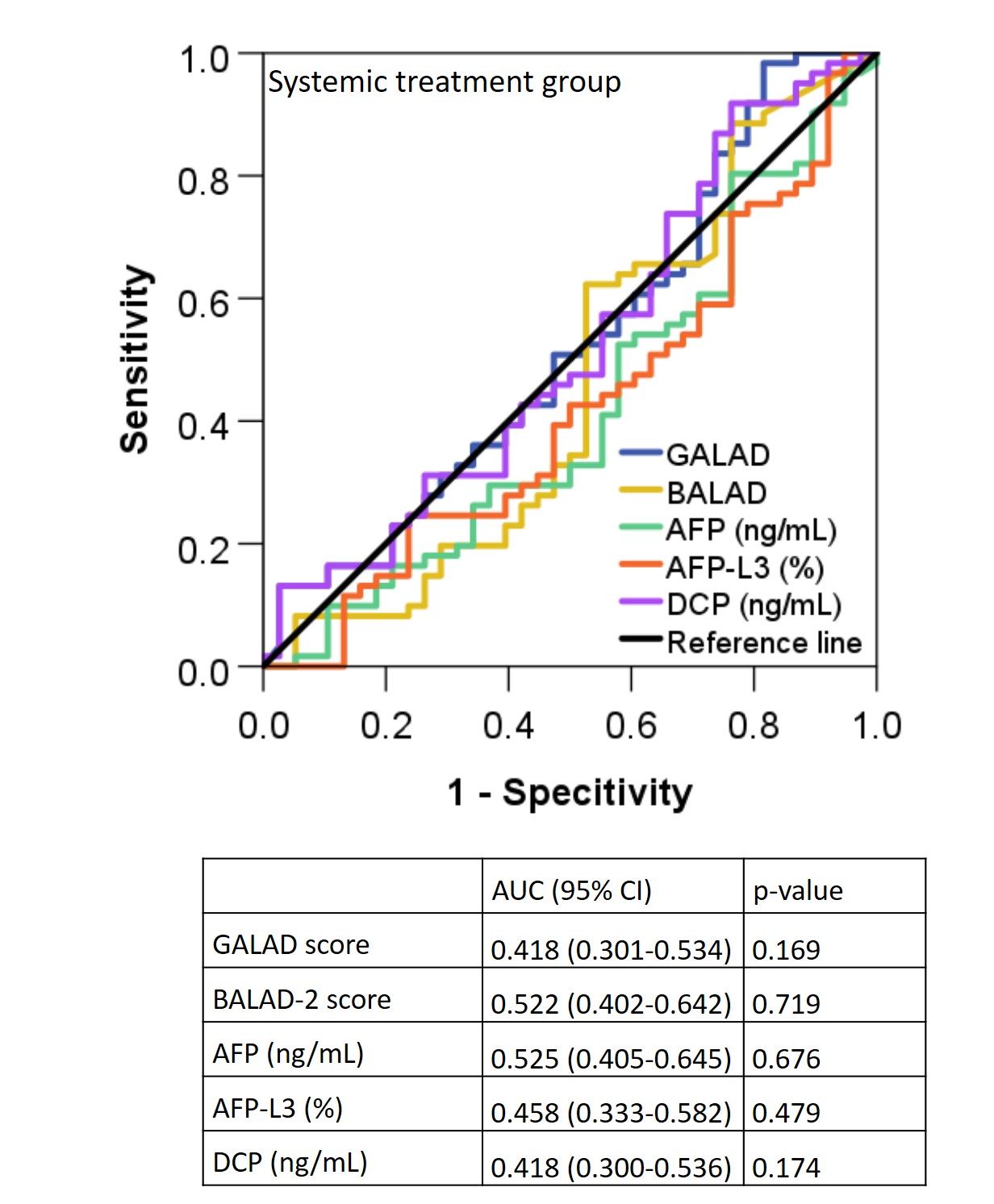

Supplement: Supplementary file 2 — Supplementary file2 (JPG 208 KB) [file 432_2023_5526_MOESM2_ESM.jpg]
