# Supplementary material for: The GALAD score and the BALAD-2 score correlate with transarterial and systemic treatment response and survival in patients with hepatocellular carcinoma
Source: J Cancer Res Clin Oncol. 2024 Feb 6;150(2):81. doi: 10.1007/s00432-023-05526-z (PMC10847183; doi:10.1007/s00432-023-05526-z)

**The GALAD score correlates with transarterial and systemic treatment response and survival in patients with hepatocellular carcinoma**

**Supporting Information**

Anne Olbrich, Johannes Niemeyer, Hendrik Seiffert, Sebastian Ebel, Olga Gros, Florian Lordick, Dirk Forstmeyer, Daniel Seehofer, Sebastian Rademacher, Timm Denecke, Madlen Matz-Soja, Thomas Berg, Florian van Bömmel

**Supplementary Tables**

| **Table S1** Systemic treatments for HCC used in our study. | |
| --- | --- |
|  | n (%) |
| Sorafenib | 70 (71) |
| Lenvatinib | 14 (14) |
| others | 15 (15) |

| **Table S2** Pretreatment to systemic treatment. | |
| --- | --- |
|  | n (%) |
| non | 26 (26) |
| TACE | 24 (24) |
| TARE | 7 (7) |
| Different Pretreatments | 40 (40) |

**Supplementary Figures**

**Supplementary Figure S1**


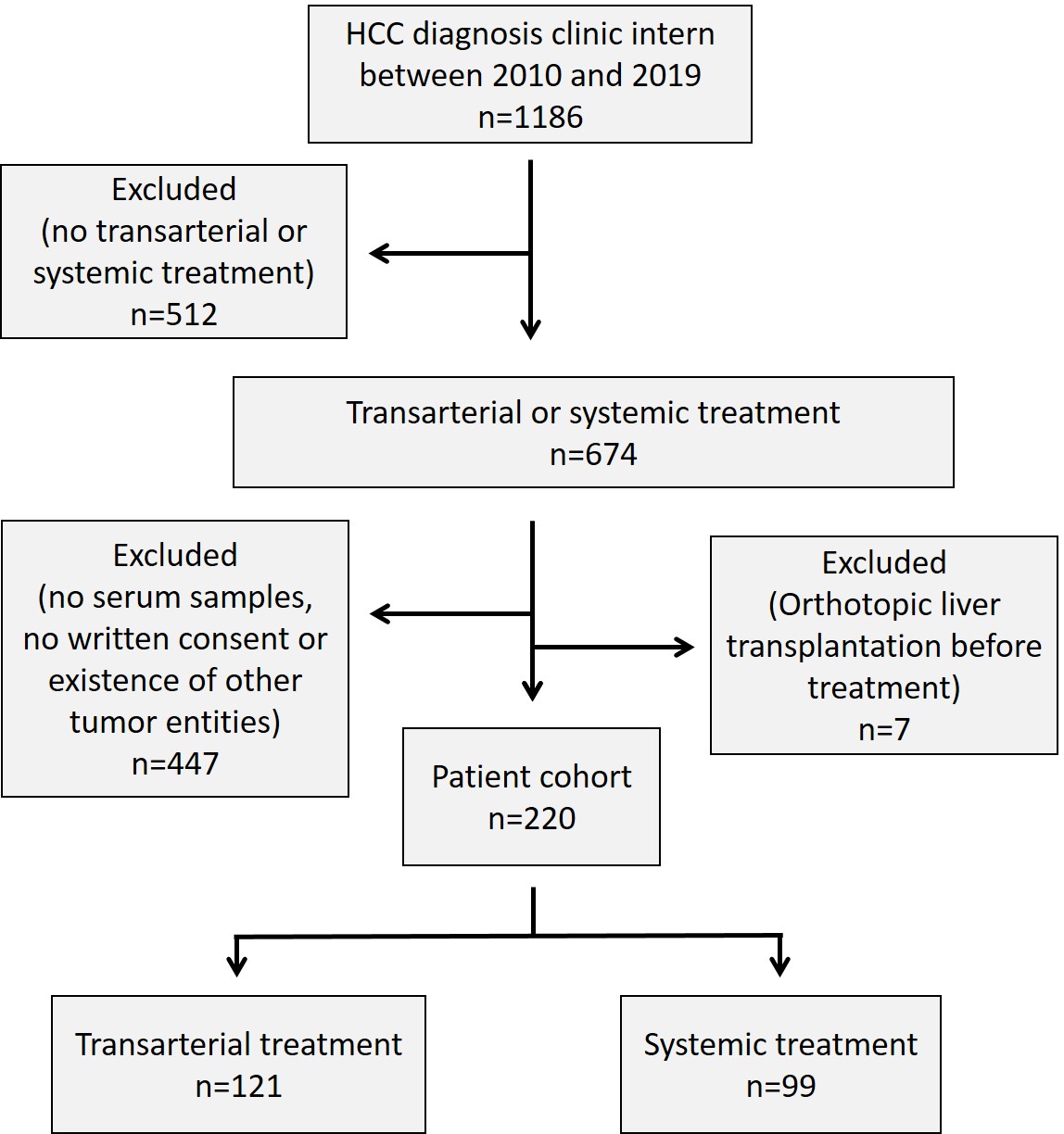


Fig. S1 Selection of the study population.

n=number of patients

**Supplementary Figure S2**

**Fig S2 Association of the GALAD score and the BALAD-2 score with survival in patients with systemic treatment.** The GALAD score and the BALAD-2 score at baseline are not associated with survival in the overall patient cohort receiving systemic treatment.


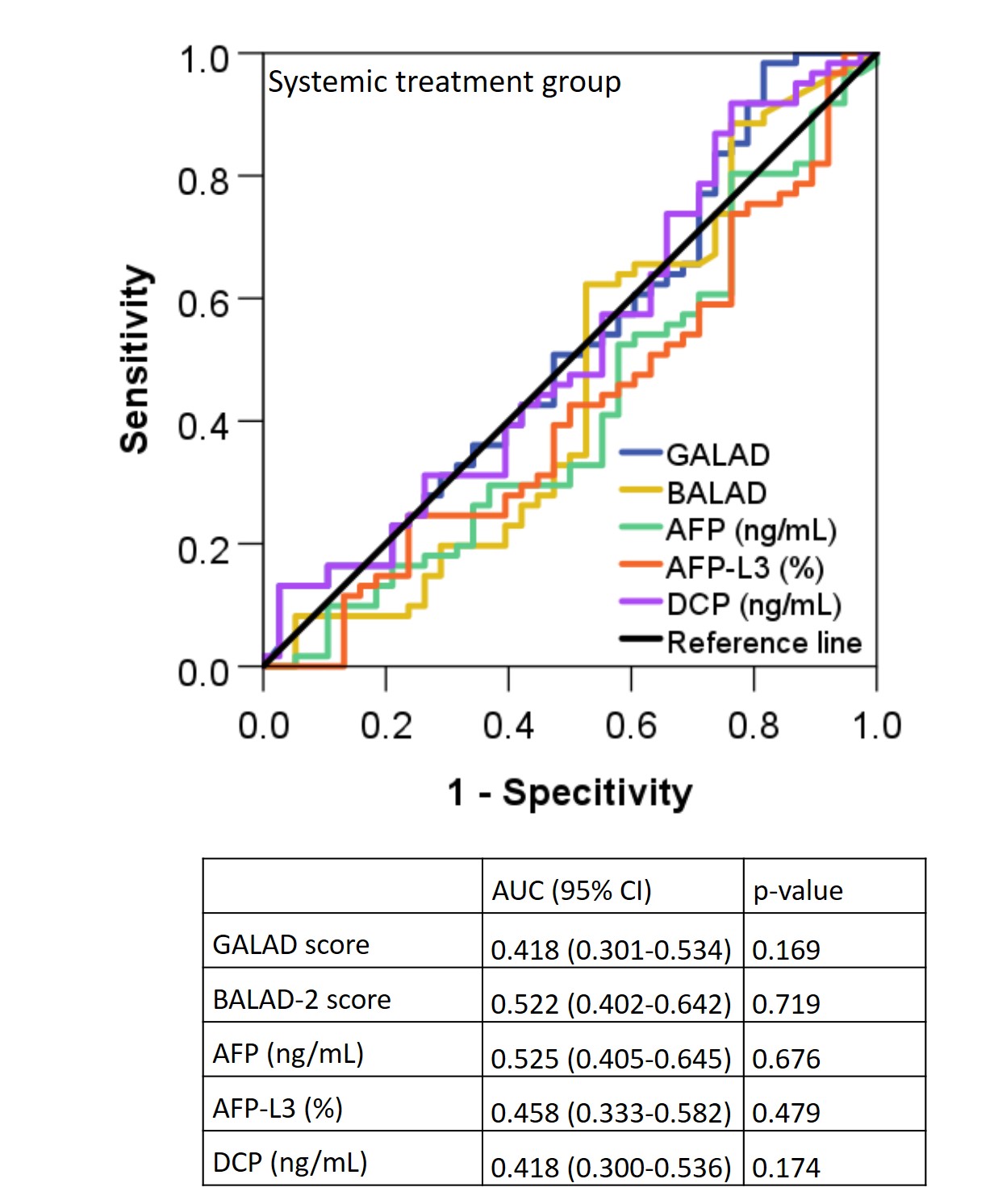

Supplement: Supplementary file 3 — Supplementary file3 (DOCX 578 KB) [file 432_2023_5526_MOESM3_ESM.docx]
